# Relationship between ultrasound detected tendon abnormalities, and sensory and clinical characteristics in people with chronic lateral epicondylalgia

**DOI:** 10.1371/journal.pone.0205171

**Published:** 2018-10-24

**Authors:** Vijayakumar Palaniswamy, Shu-Kay Ng, Nagarajan Manickaraj, Michael Ryan, Michael Yelland, David Rabago, Leanne Bisset

**Affiliations:** 1 Menzies Health Institute Queensland, Griffith University, Gold Coast, QLD, Australia; 2 Simon Fraser University, Burnaby, British Columbia, Canada; 3 School of Medicine and Public Health, University of Wisconsin, Madison, Wisconsin, United States of America; Bern University of Applied Science, SWITZERLAND

## Abstract

**Objective:**

To investigate the relationship between tendon structural changes determined by static ultrasound images (US) and sensory changes using quantitative sensory testing (QST), and clinical measures in lateral epicondylalgia.

**Materials and methods:**

Both elbows of 66 adult participants with a clinical diagnosis of lateral epicondylalgia were investigated. Using a standardised ultrasound image rating scale, common extensor hypoechogenicity, heterogenicity, neovascularity, and bony abnormalities at the enthesis were scored, and tendon thickness (longitudinal and transverse plane) was measured by a trained assessor. Sensory measures of pressure, heat and cold pain thresholds and vibration detection threshold were recorded. Pain and function were assessed using the patient-rated tennis elbow (PRTEE), pain-free grip strength, pain visual analog scale (PVAS) and quality of life (EuroQoL EQ -5D). Univariate and multivariate linear regression analyses were used to explore the association between tendon structural, sensory and clinical variables which were adjusted for age, gender and duration of symptoms.

**Results:**

A negative correlation was identified between the presence of neovascularity and cold pain threshold (P = 0.015). Multiple regression analyses revealed that a combination of female gender (P = 0.044) and transverse tendon thickness (P = 0.010) were significantly associated with vibration detection threshold in affected elbows, while gender (P = 0.012) and total ultrasound scale score (P = 0.024) were significantly associated with heat pain threshold and vibration detection threshold in unaffected elbows. Heat pain threshold and gender were significantly associated with pain and disability (PRTEE; P < 0.001), and pain-free grip strength (P < 0.001) respectively, in the affected elbows.

**Conclusion:**

Generally, structural and sensory measures were weakly correlated. However, neovascularity and transverse tendon thickness may be related to sensory system changes in LE.

## Introduction

Lateral epicondylalgia (LE), also known as tennis elbow, is a chronic tendinopathy involving the common extensor tendon. It is characterised by lateral elbow pain on resisted wrist and finger extension, focal tenderness on palpation and functional decline [1–3]. Estimates of prevalence suggest that 1–3% of working adults aged between 34 to 64 years, 50% of tennis players [4–6], smokers, and manual workers are affected by LE, with equal risk in both men and women [2, 3]. LE is recognised as a challenging condition to treat [1] and is commonly associated with a heavy economic burden due to high treatment costs, work absence and productivity loss [7].

Historically, it was believed that the clinical presentation of chronic pain in tendinopathic conditions such as LE was associated with local tendon structural changes [8, 9]. Greyscale and colour Doppler ultrasound (US) imaging are common methods used for assessing tendon structural changes including thickening, hypoechogenicity, fibrillar echotexture, neovascularity and bony abnormalities at the tendon insertion [10–12]. However, recent pathophysiological models on tendinopathy populations suggest altered sensory processing may also play a role in modulation of pain and functional impairments in tendinopathy [13–19].

Recent evidence suggests discordance between tendon structural changes identified on imaging (e.g., US, magnetic resonance), and pain and functional impairments [16, 17, 20] This discordance between structure and clinical severity may be explained by sensory system changes that lead to widespread hyperalgesia [18, 21, 22]. Widespread hyperalgesia, measured using quantitative sensory testing (QST), has been associated with increased clinical severity [17, 21, 22]. Several studies have identified sensory changes in LE, including mechanical (pressure pain threshold, PPT) and thermal (heat and cold pain threshold) hyperalgesia [17, 21, 23–25], impaired vibration detection threshold (VDT) [24], and heightened nociceptive withdrawal reflex [26]. However, there is variability in the anatomical distribution of mechanical (i.e., local vs widespread reduction in PPT) [21, 23, 26, 27] and thermal hyperalgesia (i.e., unilateral vs bilateral increase in cold pain threshold, CPT) [23, 25, 27, 28] reported in previous studies. As such, the role peripheral and/or central mechanisms may play in differentiating the clinical severity of LE has not yet been fully elucidated.

Currently, there is no conclusive model explaining the transition from localised to widespread sensory changes in LE. In LE, the tendon of the extensor carpi radialis brevis muscle is generally considered as the primary source of nociception [25, 29] and it can be hypothesised that increased nociceptive stimulation from the progressive pathological tendon structural changes results in upregulation in the neuronal input leading to widespread hyperalgesia [22, 30]. However, there is currently no evidence for the association between pathological tendon structural and sensory changes to support the hypothesis. It is proposed that evaluation of the local pathological structural changes (location of primary nociception) is paramount to understanding the transition from local to widespread hyperalgesia in individuals with varying musculoskeletal pain severity (e.g., mild, moderate and severe) [22, 30].

Therefore, the primary aim of this study was to examine the association between tendon structural and sensory characteristics in people with chronic LE. A secondary aim was to determine the interrelationship between structural, sensory, pain and functional measures. We hypothesised that tendon structural changes would be related to sensory changes in LE, and that a combination of sensory and structural characteristics will explain the heterogeneity of clinical severity in LE.

## Materials and methods

### Participants

This cross-sectional study investigates the association between several dependent and independent variables. Sixty-six individuals with a clinical diagnosis of LE were recruited between September 2013 and June 2014 from the general community. Inclusion criteria were participants aged between 18 and 75 years with a clinical diagnosis of LE [31], based on lateral elbow pain present for a minimum of six weeks that was aggravated by palpation, gripping, and resisted wrist/finger extension. In addition, a minimum score of 20/100 on the Patient-Rated Tennis Elbow Evaluation (PRTEE) was also required. All exclusion criteria were assessed on the basis of a self-report by the study participants using a standardised medical screening form. Exclusion criteria comprised of current pregnancy or breast feeding, presence of peripheral nerve involvement or cervical radiculopathy, systemic disorders including diabetes or rheumatoid arthritis, concomitant neck or other arm pain preventing usual work or recreation or required treatment within the past three months, evidence of other primary sources of lateral elbow pain such as osteoarthritis, sensory disturbance in the affected hand, history of upper limb dislocations, fractures or tendon ruptures within the preceding 10 years, history of corticosteroid injection to the affected elbow within the previous three months, or history of elbow surgery or malignancy. We performed a two-stage screening process comprising of a telephone interview followed by a standardised clinical examination by an experienced musculoskeletal physiotherapist with post-graduate training. In addition, volunteers completed a medical screening questionnaire prior to the clinical examination, which included questions regarding any history of systemic conditions or other co-morbidities. The physiotherapist discussed the results of the screening assessments with the co-investigator (MY), who is an experienced musculoskeletal doctor. Volunteers who were suspected of having co-morbidities such as osteoarthritis of the elbow joint, were excluded from the study. The study was conducted in accordance with the Declaration of Helsinki and written informed consent was obtained from all participants prior to their participation. Ethical approval was obtained from Griffith University Human Research Ethics Committee.

### Sample size calculation

A priori analysis using the G* Power 3.1 statistical software [32] was performed to calculate the required sample size based on the following parameters: multiple linear regression fixed model (R2 deviation from zero) with 13 factors, statistical significance level of 0.05, large effect size (0.35) and 80% power [32]. The results of the a priori analysis revealed that a sample size of N = 64 was required for the study.

### Outcome measures

#### Clinical measures

All clinical measurements were performed by two experienced investigators (MR and NM) with 10 to 17 years’ clinical experience. Self-reported pain and functional disability were quantified using the validated and reliable (ICC 0.93) PRTEE [33]. Pain intensity at rest and worst imaginable pain experienced in the preceding week was documented on a reliable (ICC = 0.89) 100 mm visual analog scale (PVAS: 0 = no pain, 100 = worst pain imaginable) [34]. Quality of life was measured using the reliable (ICC 0.87) EuroQoL EQ-5D [35]. A Digital Analyzer grip dynamometer (MIE Ltd, Leeds UK) was used to quantify pain-free grip (PFG) on affected and unaffected sides. With the test arm in elbow extension and pronation, participants were instructed to gradually squeeze the dynamometer and stop when the first sensation of pain was perceived (maximal grip force was measured on the unaffected side) [36, 37]. PFG was measured three times with a 1-min rest interval between each measurement, and the average value was then used in further analyses. PFG (N) is a highly reliable (ICC > 0.97) measure in assessing disability in LE [36]. In addition to the clinical measures, participant’s characteristics including age, gender, occupation, duration of symptoms and hand dominance were also documented.

#### Quantitative sensory testing

Quantitative sensory testing (QST) is a suite of non-invasive psychophysical tests used to investigate hyper-function of small diameter sensory fibers and hypo-function of large diameter sensory fibers [38]. Measurement of thermal pain threshold can be useful in the evaluation of C and A-delta nerve fiber function. These small fibers mediate both non-pain and painful thermal sensation and activating the spinoreticulothalamic tracts centrally [38]. QST is reported to be useful in evaluating impairments of the sensory system in several musculoskeletal conditions such as whiplash injury, repetitive strain injury, lateral epicondylalgia [39, 40] and knee osteoarthritis. A trained investigator assessed pressure pain threshold (PPT), cold pain threshold (CPT), heat pain threshold (HPT), and vibration detection threshold (VDT) [38, 41].

#### Pressure pain threshold

PPT was measured over the common extensor tendon insertion on both affected and unaffected elbows using a digital pressure algometer (Somedic AB, Fasrsta, Sweden) and a standardised testing protocol consistent with previous studies [39]. The assessor applied pressure at a constant rate (40kPa/sec), with the probe (area of 1 cm2) perpendicular to the skin [39]. The amount of force (kPa) required to evoke the first sensation of pain, distinct from pressure, was recorded, and three consecutive measurements with a rest time of 30 seconds between trials were averaged and used in further analyses. High reliability was established in a previous study using this equipment (ICC 0.99) [42].

#### Thermal (heat and cold) pain thresholds

HPT and CPT were measured bilaterally over the lateral elbow using the method of limits in accordance with the German Network of Neuropathic Pain guidelines [43]. All participants were tested in a quiet room at a standard temperature (24°C) using the Thermosensory Analyser (TSA-II, Medoc, Ramat-Yishai, Israel). A thermode (30 x 30 mm), placed over the lateral elbow, delivered either a cold or heat stimulus at a constant rate of 1°C/sec from a baseline temperature (32°C) until a pain sensation was first perceived by the participant or when the upper limit of 50°C or lower limit of 0°C is reached. The participants were asked to press a response button when they first perceived a painful heat or cold sensation. The temperature corresponding to the point of termination was documented as HPT or CPT, and the average of three recorded HPT and CPT pain threshold measurements were used in further analyses. High inter-rater reliability has been reported for both HPT (ICC 0.87) and CPT (ICC 0.89) using this equipment [42].

#### Vibration detection threshold (VDT)

Each participant was seated comfortably with the test hand supported on a table. A computer-controlled Vibratory Sensory Analyser VSA-3000 (Medoc Ltd, Ramat Yishai, Israel) with a stimulating area of 1.2 cm2 was utilised to measure VDT of the distal phalanx of the middle finger. The vibration stimulus was progressively increased at a constant rate from 0.1 to 130 μm/s, and the participant pressed the response button as soon they first perceived the vibration. Five measurements were made on each hand, and the mean was used in further analyses. A previous study observed high reliability for measuring vibration sensation (ICC 0.86) [44].

#### Ultrasound imaging

All ultrasound imaging on bilateral elbows were performed immediately following the clinical examination, by an experienced examiner (MR) with five years of musculoskeletal US experience. Ultrasound imaging was performed using SonixTouch US equipment (Ultrasonix, Richmond, BC, Canada) [45] with a high frequency 38 mm linear transducer (L14–5W/60) and a frequency range of 14–5 MHz. Firstly, greyscale US with standardised B-mode image settings (depth 2cm, gain 55%, dynamic range 68DB, map 4, power 0) was performed over the common extensor tendon, with participants in a seated position and the arm resting on the table in approximately 70° shoulder abduction, 90° elbow flexion and pronated forearm [10, 46, 47]. Following the greyscale US imaging, the presence of neovascularity within the same region was evaluated by standardised power Doppler US settings (pulse repetition frequency of 500 Hz, wall filter 40 Hz, gain range of 55–90%) with minimal probe pressure and adjusted sensitivity to detect low flow and minimal noise level. Longitudinal and transverse plane static images of the common extensor tendon were obtained with the transducer positioned parallel to the long axis of the tendon with the head of the radius and contour of the lateral epicondyle as anatomical reference guides. All the captured images were stored with a unique identifier in a re-identifiable format, in a password-protected storage drive.

#### Image analysis

All the captured ultrasound images were evaluated by an independent trained single assessor (VP) who was blind to clinical and sensory outcomes, as well as to the affected side. The assessor (VP) was not involved in the collection of US images. The assessor underwent targeted training for six months from an experienced (>10 years) musculoskeletal ultrasonographer in the acquisition, grading and measurement of US images. Following the training phase, the assessor and the ultrasonographer practised the US image grading procedure on 15 de-identified elbow images to establish a minimum of 80% overall agreement in using the US grading system. In addition, a preliminary validation study assessing the reliability between a musculoskeletal radiologist (gold standard) and non-radiologist (VP) revealed excellent (ICC > 0.8) inter-rater reliability between assessors in grading tendon abnormalities such as hypoechogenicity, heterogenicity, and neovascularity, and in measuring tendon thickness. There was moderate inter-rater reliability (ICC = 0.5) for identifying bony abnormalities and poor inter-rater reliability (ICC < 0.4) for identifying the presence of intratendinous calcification on recorded US images. Therefore, following a consensus meeting with the radiologist and the musculoskeletal ultrasonographer, the presence of intratendinous calcification was removed from the final scoring scale due to this poor reliability. Previous studies have reported acceptable test-retest reliability (ICC > 0.7) for grading greyscale and colour Doppler findings by non-radiologists using the semi-quantitative grading method [47, 48].

#### Ultrasound assessment of tendon abnormalities

Tendon echotexture and vascularity on the captured US images of bilateral elbows were graded with respect to focal areas of hypoechogenicity, diffuse heterogeneous areas, neovascularity and insertional bony abnormalities. For the purpose of grading, US images representing anterolateral, central (middle) and posterior region of the common extensor tendon exhibiting intratendinous abnormalities and vascularity were examined by the assessor [49]. Hypoechogenicity and hypervascularity were graded as none, mild, moderate or severe (scored 0 to 3 respectively) [10]. In the event of a significantly uneven distribution of ultrasound grades for hypoechogenicity and neovascularity, the 4-point scales were each dichotomised into a 2-point scale and used in the sub-grouping analyses. That is, hypoechogenicity grades 0 to 2 were subgrouped into a ‘mild’ category with grade 2 labelled as a ‘severe’ category, and neovascularity grade 0 was labelled as an ‘absent’ category and grades 1 to 3 were labelled as ‘present’. The absence or presence (score 0 and 1 respectively) of diffuse heterogeneous areas and bony abnormalities were scored dichotomously. The sum of all features (excluding the dichotomised hypoechoic and hypervascular scores) gave a total ultrasound scale score (TUSS, maximal score = 8; Table 1) [10–12, 47, 50].

**Table 1:** Total ultrasound image rating scale.

Ultrasound feature	Description	Grading range	Grading criteria	Maximum score
Hypoechogenicity	Ordinal	0 to 3	0 = normal fibrillar & hypoechoic structure	3
			1 = hypoechoic lesions affecting less than 30% of whole section of the tendon.	
			2 = hypoechoic lesions affecting more than 30% and less than 50% of the whole section of the tendon.	
			3 = single large or multiple hypoechoic lesions affecting more than 50% of the whole section of the tendon / high-grade tendinosis.	
Neovascularity	Ordinal	0 to 3	0 = no detectable neovessels	3
			1 = neovessels detected in less than 30% of the whole section of the tendon	
			2 = neovessels detected in more than 30% but less than 50% of the whole section of the tendon	
			3 = neovessels detected in more than 50% of the whole section of the tendon	
Heterogeneity	Dichotomous	1 or 0	1 = presence, 0 = absence	1
Bony abnormalities	Dichotomous	1 or 0	1 = presence, 0 = absence	1

**Total Ultrasound Scale Score**				8

#### Ultrasound assessment of tendon thickness

**Fig 1:**
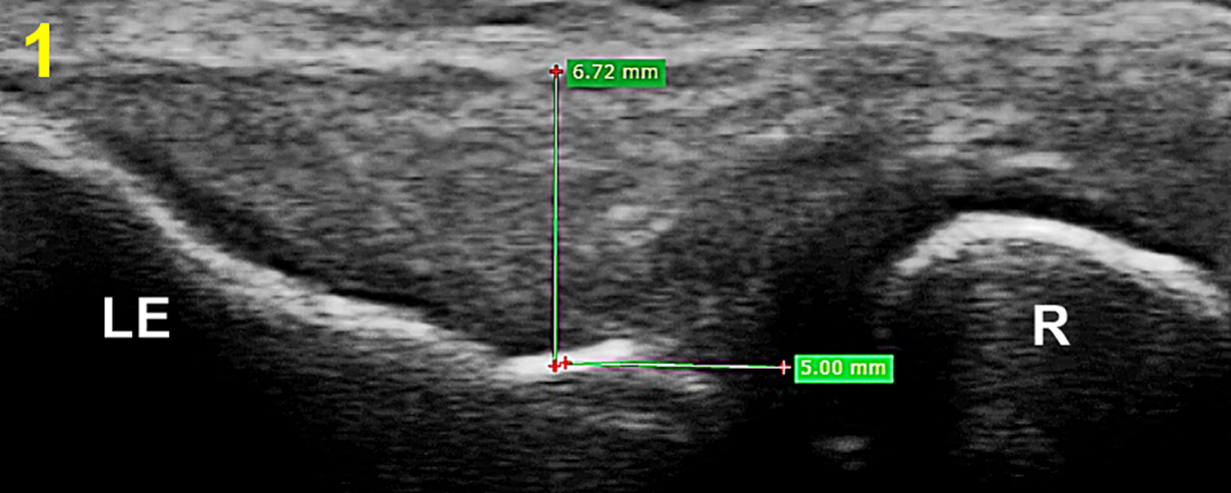
Longitudinal ultrasound view demonstrating thickness measurement (mm) of the common extensor tendon. LE: lateral epicondyle, R: radius.

**Fig 2:**
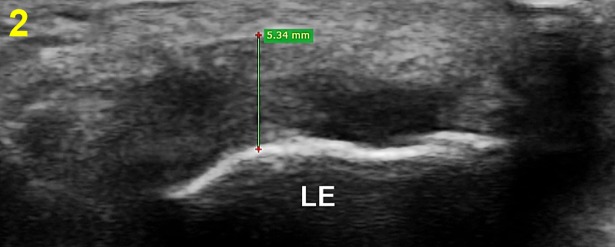
Transverse ultrasound view demonstrating thickness (mm) measurement of the common extensor tendon. LE: lateral epicondyle, R: radius.

The thickness of the common extensor tendon was measured on the longitudinal and transverse images considered to show optimal anatomical details of the tendon using the length measuring tool of the RadiAnt DICOM Viewer V.3.4. 1 (Mediexant, Poznan, Poland) [50]. Utilising a 5 mm distance from the radiohumeral joint margin as the standard reference point, longitudinal image tendon thickness was measured as the perpendicular distance between the tendon surface and the cortical bony interface of lateral epicondyle (Fig 1) [10, 47, 51]. Transverse image tendon thickness was assessed by measuring the perpendicular distance from the capitellum to the superficial tendon boundary (Fig 2). High reliability (ICC > 0.7) for measuring common extensor tendon thickness on captured images has been reported in previous studies using this standardised approach [47, 51].

### Statistical analysis

All statistical analyses were performed using SPSS Statistics version 24 (IBM, Chicago, IL, USA). Descriptive statistics for continuous data were expressed as means ±standard deviations while categorical data were reported as frequencies (percentages). Normality assumptions of all continuous variables were assessed using Shapiro-will test statistics. In order to assess differences between affected and unaffected sides, paired samples t-test, Wilcoxon signed rank, McNemar’s test, and Friedman’s statistics were used for normally distributed continuous variables, non-normally distributed continuous variables, dichotomous and ordinal variables respectively. Participants who reported bilateral symptoms were excluded from analyses comparing affected and unaffected sides.

To assess differences in clinical and sensory measures for grades of hypoechogenicity, neovascularity and bony abnormalities, one-way analyses of variance (ANOVA) were used. Univariate and multivariate linear regression analyses were then used to examine the relationship between tendon structural and sensory measures for both affected and unaffected elbows in a two-stage procedure as described in a previous study [52]. In these analyses, potential confounders including demographic characteristics of age, gender, duration of condition, and history of corticosteroid injection were also assessed as factors in the regression analyses, due to their known predictive value for pain and disability (PRTEE), PFG [27, 52], and potential impact on tendon structure [53] In order to provide meaningful and important associated variables for consideration in the final multiple regression analysis model, univariate linear regression analyses were initially performed for each of the potentially associated variables at a statistical significance of P < 0.15 [54]. The significant variables (P < 0.15) identified from the univariate analyses were then examined in a final multivariate regression analysis model using backwards elimination approach to determine the most parsimonious model that described the relationship among variables [54]. To ensure the validity of results, additional analyses were performed to substantiate the findings from the multivariate regression analyses; 1) forward stepwise regression tests were performed to ascertain whether the significant associated factors identified in the final model were similar to the “backward” stepwise regression method, and 2) separate correlation analyses were performed to determine whether collinearity or multicollinearity existed between variables. Further, ordinal variables (hypoechogenicity, hypervascularity) were treated as numerical variables for both univariate and multivariate regression analyses. Secondary analysis included a similar statistical approach to determine the significant structural and sensory measures associated with PRTEE and PFG of affected elbows and PFG measures of unaffected elbows respectively. The significance level was set at 5% with two-tailed hypothesis test for all analyses except the univariate linear regression analyses.

## Results

### Participant characteristics

Descriptive statistics for the demographic, tendon structural, sensory and clinical measures of participants are presented in Table 2. Bilateral symptoms were reported by 10 (15.2%) participants. Ultrasound imaging was not performed on the unaffected elbow for one participant who reported unilateral LE symptoms. Nine participants (13.6%) had a previous corticosteroid injection greater than six months ago. In the affected elbows, the presence of grade 3 (72.7%) hypoechogenicity, absence of neovascularity (81.8%) and presence of insertional bony abnormalities (74.2%) were the most prevalent ultrasound findings, whereas the presence of grade 2 and 3 (44.6% each) and absence of neovascularity (87.5%) were the most common ultrasound findings in the unaffected elbows of our LE participants.

**Table 2:** Demographic, clinical, tendon structural and sensory measures for the affected (N = 66) and unaffected elbows (N = 56).

Demographic characteristics	Mean ±SD; n (%)		
Age, years	50.5 ±7.6		
Body mass index, kg/m^2^	26.7 ±3.9		
Occupation, N			
Manual	29 (43.9%)		
Non-manual	32 (48.5%)		
Not working	5 (7.6%)		
Male, N	41 (62.1%)		
Dominant side affected, N	43 (65.2%)		
Right side dominant, N	60 (90.9%)		
**Clinical measures**			
Duration of symptoms, weeks	50.8 ±2.9		
Previous injection treatment >3 months, N	9 (13.6%)		
PRTEE total score, /100	30.8 ±10.3		
PRTEE pain, /50	22.6 ±6.5		
PRTEE function, */50*	15.2 ±8.3		
Rest pain, mm	17.1 ±16.2		
Worst pain, mm	67.1 ±22.8		
EuroQol EQ-5D, /100	82.3 ±13.7		
	**Affected side**	**Unaffected side**	**Affected—Unaffected mean difference (95% CI)**
Pain-free grip strength, Newtons	151.0 ±97.9	263.1.6 ±105.3*	-106.6 (-135.3, -77.9)
**Tendon structural measures**			
Longitudinal tendon thickness, mm	6.1 ±0.8	5.5 ±0.7*	0.5 (0.3, 0.7)
Transverse tendon thickness, mm	5.5 ±0.6	4.8 ±1.4*	0.7 (0.2, 1.2)
Hypoechogenicity, N			
Grade 0	-	-	
Grade 1	2 (3.0%)	6 (10.7%)*	
Grade 2	16 (24.2%)	25 (44.6%)*	
Grade 3	48 (72.7%)	25 (44.6%)*	
Neovascularity, N			
Grade 0	54 (81.8%)	49 (87.5%)	
Grade 1	12 (18.2%)	9 (12.5%)	
Grade 2	-	-	
Grade 3	-	-	
Presence of bony abnormalities, N	48 (72.7%)	29 (51.8%)*	
TUSS /8	4.6 ±0.9	3.9 ±1.1*	
**Sensory measures**	**Affected side (n = 66)**	**Unaffected side (n = 56)**	
PPT, kPa	254.8 ±107.2	371.7 ±136.7*	-113.3 (-144.8, -81.9)
CPT, °C	9.3 ±10.9	7.4 ±9.3*	2.2 (0.3, 4.1)
HPT, °C	46.6 ±3.4	47.5 ±2.1*	-0.8 (-1.5, -0.09)
VDT, μm/s	1.8 ±1.1	1.7 ±1.5	0.1 (-0.2, 0.4)

*significant differences between affected vs unaffected elbows *P* ≤ 0.05, PPT: pressure pain threshold, HPT: heat pain threshold, CPT: cold pain threshold, VDT: vibration detection threshold, TUSS: total ultrasound scale score, PRTEE: Patient-Rated Tennis Elbow Evaluation, EQ-5D: EuroQol EQ-5D quality of life questionnaire.

### Within-subject differences

The affected side exhibited significantly higher TUSS scores, greater tendon thickness, a greater number of participants with severe hypoechogenicity, reduced PFG, PPT, and HPT and greater CPT compared to the unaffected side (Table 2). The presence of heterogeneous fibrillar echotexture was observed in 100% of affected elbows. As such, this outcome was unable to be included in further statistical analyses as it was observed to be a constant variable (i.e., has just one value: the presence of heterogeneous echotexture = 1).

### Comparison of sensory and clinical findings between US grades for structural abnormalities

Sensory and clinical characteristics of participants are reported in Table 3. CPT was significantly superior in individuals identified with neovascularity in the affected elbows. In contrast, resting pain (mm) was worse among participants identified with insertional bony abnormalities in the affected elbows. For the unaffected elbows, VDT was significantly worse in individuals with insertional bony abnormalities.

**Table 3:** Mean ±SD of clinical and sensory measures for dichotomised greyscale ultrasound outcomes for the affected (N = 66) and unaffected side (N = 56).

US parameter	Duration of condition, weeks	PRTEE, /100	Resting pain, /100	Worst pain, /100	PFG, Newtons	EQ-5D, /100	PPT, kPa	HPT, °C n = 66 (AF) n = 56 (UAF)	CPT, °C n = 66 (AF) n = 56 (UAF)	VDT, μm/s n = 66 (AF) n = 56 (UAF)
**Hypoechogenicity**										
**Mild**										
AF, n = 18	52.0 ±61.8	30.6 ±8.3	13.3 ±15.7	67.7 ±21.2	135.2 ±83.2	81.1 ±10.6	270.8 ±104.8	46.0 ±3.3	11.7 ±11.0	1.6 ±0.8
UAF, n = 30	-	-	-	-	239 ±97.9	-	364.5 ±126.2	47.3 ±3.4	6.6 ±9.4	1.3 ±0.9
**Severe**										
AF, n = 48	35.8 ±45.9	30.9 ±10.2	18.5 ±16.3	66.8 ±23.6	157.0 ±103.1	82.8 ±14.3	248.2 ±108.7	46.8 ±3.4	8.4 ±10.9	1.6 ±1.2
UAF, n = 25	-	-	-	-	291.3 ±108.8	-	380.4 ±150.7	47.7 ±2.4	8.1 ±9.2	1.9 ±1.3
**Neovascularity**										
**Absent**										
AF, n = 54	40.3 ±54.0	31.1 ±10.6	17.4 ±15.6	67.4 ±20.1	141.6 ±95.2	80.9 ±13.9	252.8 ±108.8	46.3 ±3.6	10.8 ±11.4	1.5 ±1.2
UAF, n = 48	-	-	-	-	255.6 ±101.4	-	383.8 ±141.5	47.5 ±2.1	7.1 ±8.9	1.6 ±1.1
**Present**										
AF, n = 12	39.5 ±34.5	29.7 ±7.1	15.8 ±19.2	65.8 ±29.9	192.5 ±103.2	89.0 ±7.8	264.2 ±104.0	48.1 ±1.8	2.4 ±3.9*	1.7 ±0.8
UAF, n = 7	-	-	-	-	314.4 ±124.9	-	292.1 ±57.8	47.4 ±2.6	8.6 ±12.4	1.5 ±1.1
**Insertional bony abnormalities**										
**Absent**										
AF, n = 18	37.2 ±44.7	27.5 ±7.4	8.8 ±14.0	64.4 ±21.2	162.9 ±93.9	78.8 ±11.5	248.9 ±117.3	46.4 ±3.4	8.8 ±10	1.4 ±0.8
UAF, n = 27	-	-	-	-	234.9 ±101.3	-	354.0 ±126.6	47.1 ±2.0	7.0 ±9.6	1.2 ±0.7
**Present**										
AF, n = 48	41.3 ±53.2	32.1 ±10.2	20.2 ±16.0*	68.1 ±23.5	146.4 ±99.3	83.7 ±13.9	257.2 ±104.1	46.7 ±3.4	9.5 ±11.2	1.6 ±1.2
UAF, n = 29	-	-	-	-	288.4 ±104.0	-	390.0 ±146.6	47.9 ±2.1	7.5 ±9.0	1.9 ±1.3*

* significant difference between US sub-groups *P* < 0.05.

US: ultrasound, PRTEE: Patient-Rated Tennis Elbow Evaluation, PFG: pain-free grip strength, EQ-5D: EuroQol EQ-5D quality of life questionnaire, PPT: pressure pain threshold, HPT: heat pain threshold, CPT: cold pain threshold, VDT: vibration detection threshold, AF: affected side, UAF: unaffected side.

### Relationship between demographic, structural and sensory measures

The association between demographic, structural and sensory measures in affected and unaffected elbows are reported in Tables 4 and 5, respectively. Neovascularity on the affected side was the only variable to show a significant association with HPT (P = 0.08) at the univariate level. Similarly, gender and neovascularity were the only significant variables associated with PPT on the affected (P = 0.11) and unaffected side (P = 0.09) respectively (Tables 4 and 5), so no further analyses were performed. No significant correlation was found between the age, duration of symptoms, history of corticosteroid injection treatment and tendon structural and sensory measures.

**Table 4:** Univariate and multivariate regression analyses examining variables associated with sensory measures for the affected side.

	HPT, °C Univariate	PPT, kPa Univariate	CPT, °C Univariate	CPT, °C Multivariate	VDT, μm/s Univariate	VDT, μm/s Multivariate
Variables	β (95% CI) *P*^*†*^	β (95% CI) *P*^*†*^	β (95% CI) *P*^*†*^	β (95% CI) *P*	β (95% CI) *P*^*†*^	β (95% CI) *P*
Intercept				10.8 (8.0, 13.7) <0.001		2.8 (-0.2, 7.6) 0.25
Age	-0.02 (-0.1, 0.08) 0.60	0.1 (-3.5, 3.8) 0.94	0.05 (-0.3, 0.4) 0.77		0.03 (-0.02, 0.09) 0.06^**†**^	
Female gender	-0.7 (-2.4, 0.9) 0.39	-44.5 (-99.7, 10.7) 0.11^**†**^	1.6 (-3.9, 7.3) 0.54		-0.7 (-1.3, -0.1) 0.01^**†**^	-0.6 (-1.3, -0.02) 0.04*
Duration	0.0 (-0.01, 0.01) 1.0	-0.2 (-0.8, 0.2) 0.31	-0.01 (-0.05, 0.03) 0.61		-0.005 (-0.01, 0.001) 0.08^**†**^	
Hypoechogenicity	0.5 (-1.1, 2.1) 0.49	-24.7 (-76.0, 26.4) .33	-2.6 (-7.8, 2.5) 0.31		0.02 (-0.5, 0.6) 0.94	
Neovascularity	1.6 (-0.2, 3.3) 0.08^**†**^	-11.4 (-60.4, 83.2) .75	-8.4 (-15.1, -1.6) .01^**†**^	-8.4 (-15.1, -1.6) 0.015*	0.1 (-0.6, 0.9) 0.67	
Bony abnormalities	0.2 (-1.6, 2.2) 0.76	8.2 (-52.1, 68.7) 0.78	0.7 (-5.4, 6.9) 0.82		0.2 (-0.5, 0.8) 0.59	
LTT	0.4 (-0.5, 1.4) 0.40	-4.6 (-36.9, 27.6) 0.77	-1.4 (-4.6, 1.7) 0.37		0.3 (-0.6, 0.6) 0.11^***†***^	
TTT	0.3 (-1.2, 1.8) 0.69	-28.9 (-79.1, 21.1) .25	-1.0 (-5. 6, 3.6) 0.67		0.8 (0.3, 1.3) <0.001^***†***^	0.6 (0.1 to 1.2) 0.01*
TUSS	0.5 (-0.3, 1.4) 0.20	-4.3 (-34.5, 25.8) 0.77	-2.2 (-5.2 to 0.72) 0.13^**†**^		0.08 (-0.2, 0.4) 0.61	
Adjusted R^2^				0.075		0.225

β = Unstandardised regression coefficients

*statistically significant *P* < 0.05

CI: confidence interval

^***†***^*P* significance ≤ 0.15

HPT: heat pain threshold, PPT: pressure pain threshold, CPT: cold pain threshold, VDT: vibration detection threshold, LTT: longitudinal tendon thickness, TTT: transverse tendon thickness, TUSS: total ultrasound scale score.

**Table 5:** Univariate and multivariate regression analysis examining variables associated with sensory and PFG measures of the unaffected side for unilateral LE participants (N = 56).

Variables	HPT, °C Univariate β (95% CI) *P*	HPT, °C Multivariate β (95% CI) *P*	CPT, °C Univariate β (95% CI) *P*	VDT, μm/s Univariate β (95% CI) *P*	VDT, μm/s Multivariate β (95% CI) *P*	PPT, kPa Univariate β (95% CI) *P*	PFG, Newtons Univariate β (95% CI) *P*	PFG, Newtons Multivariate β (95% CI) *P*
Intercept		49.6 (47.9, 51.2) 0.001			-0.3(-1.9, 1.3) 0.02			433.9 (362.0, 505.9) <0.001
Age	0.02 (-0.05, 0.09) 0.52		0.09 (-0.2, 0.4) 0.57	0.04 (-0.01, 0.08) 0.01^*†*^		2.1 (-2.6, 7.0) 0.37	-0.8 (-4.4, 2.7) 0.65	
Female gender	-1.4 (-2.5, 0.3) 0.12^*†*^	-1.4 (-2.5, 0.3) 0.01*	-0.2 (-5.3, 4.8) 0.92	-0.5 (-1.1, 0.07) 0.08^*†*^		-12.8 (-89.5, 63.9) 0.73	-120.4 (-168.3, -72.5) <0.001^*†*^	-120.1 (-168.4, -72.5) <0.001*
Hypoechogenicity	0.05 (-0.8, 0.9) 0.90		1.4 (-2.2, 5.2) 0.43	0.3 (-0.1, 0.8) 0.12^*†*^		-16.0 (-42.5, 74.7) 0.58	48.5 (7.5, 89.5) 0.02^*†*^	
Neovascularity	-0.11 (-1.8, 1.6) 0.90		1.5 (-6.0, 9.1) 0.68	-0.1 (-1.0, 0.8) 0.83		-91.6 (-201.1, 17.8) 0.09^*†*^	58.7 (-25.9, 143.5) 0.17	
Bony abnormalities	0.8 (-0.3, 1.9) 0.15^*†*^		0.5 (-4.5, 5.5) 0.83	0.6 (00.5, 1.2) 0.03^*†*^		36.0 (-39.4, 111.4) 0.34	53.5 (-2.1, 109.1) 0.05^*†*^	
LTT	0.61 (-0.2, 1.4) 0.13^*†*^		-0.7 (-4.3, 2.7) 0.65	0.03 (-0.4, 0.5) 0.87		35.7 (-22.9, 94.4) 0.22	32.4 (7.5, 72.3) 0.10^*†*^	
TTT	-0.05 (-0.5, 0.4) 0.81		0.6 (-1.6, 2.9) 0.57	0.2 (-0.1, 0.6) 0.12^*†*^		4.7 (-31.1, 40.5) 0.79	3.6 (-25.0, 32.2) 0.79	
TUSS	0.4 (-0.1, 1.0) 0.10^*†*^		-0.04 (-2.4, 2.2) 0.97	0.3 (0.01, 0.6) 0.04^*†*^	0.4 (0.06, 0.8) 0.02*	8.0 (-26.0, 42.1) 0.63	37.2 (13.2, 61.2) <0. 001^*†*^	
Adjusted R^2.^		0.094			0.127			0.312

β = Unstandardised regression coefficients

*statistically significant *P* < 0.05

CI: confidence interval

^***†***^*P* significance ≤ 0.15

HPT: heat pain threshold, PPT: pressure pain threshold, CPT: cold pain threshold, VDT: vibration detection threshold, PFG: pain-free grip strength, LTT: longitudinal tendon thickness, TTT: transverse tendon thickness, TUSS: total ultrasound scale score.

The only significant variable associated with CPT on the affected side was neovascularity, which explained 7.5% of the variance in CPT. For VDT, women were more likely to have a lower threshold (women mean 1.1 ± 0.5 μm/s versus men 1.9 ± 1.3 μm/s) to vibration detection. For the unaffected side, female gender was the only significant variable associated with HPT in the final multivariate model, which explained 9.4% of the variance. Further, TUSS was significantly associated with VDT in the final model, explaining 12.7% of the variance (Table 5).

### Relationship between structural, sensory and clinical measures

The association between structural and sensory characteristics with clinical measures of pain and function are reported in Tables 5 and 6. Higher HPT (i.e., less hyperalgesic) was the only significant variable associated with higher PRTEE scores (more severe pain and disability) in the final model, explaining 10.4% of the variance in the affected elbows, while female gender was associated with lower pain-free grip strength, explaining 21.9% of the variability of pain-free grip strength on the affected side (Table 6). For the unaffected side, the final model analysis revealed that women recorded lower pain-free grip strength, explaining 31.2% of the total variance (Table 5).

**Table 6:** Univariate and multivariate regression analyses examining clinical variables associated with PRTEE and PFG in the affected side (N = 66).

Variables	PRTEE /100 Univariate β (95% CI) *P*	PRTEE /100 Multivariate β (95% CI) *P*	PFG, Newtons Univariate β (95% CI) *P*	PFG, Newtons Multivariate β (95% CI) *P*
**Demographic**				
Age	0.1 (-0.2, 0.4) 0.44		0.6 (-2.5, 3.8) 0.68	
Female gender	-0.6 (-4.3, 5.6) 0.80		-94.6 (-139.3, -49.8) <0.01	-96.6 (-142.1, -1.2) <0.01
Duration	-0.01 (-0.05, 0.03) 0.72		-0.2 (-0.6, 0.3) 0.46	
**Tendon structural measures**				
Hypoechoechogenicity	0.3 (-4.2, 4.9) 0.87		29.0 (-17.2, 74.9) 0.21	
Neovascularity	-1.3 (-7.6, 4.8) 0.66		50.5 (-11.1, 112.3) 0.10†	
Bony abnormalities	4.5 (0.7, 9.8) 0.08†		-16.4 (-70.9, 38.0) 0.54	
LTT	0.5 (-2.3, 3.3) 0.73		28.7 (0.4, 57.0) 0. 04†	
TTT	2.2 (-1.9, 6.3) 0.30		22.1 (-17.9, 62.3) 0.27	
TUSS	1.1 (-0.8, 3.1) 0.26		14.8 (-11.8, 41.5) 0.27	
**Sensory measures**				
HPT	1.0 (0.3, 1.6) <0.01†	1.03 (.32,1.74) <0.01*	0.1 (-7.7, 8.1) 0.96	
CPT	-0.2 (-0.4, 0.05) 0.13†		-0.7 (-3.0, 1.4) 0.49	
VDT	1.8 (-0.2, 4.0) 0.07†		17.2 (-4.0, 38.5) 0.11†	
PPT	-0.003 (-0.02, 0.03) 0.78		-0.1 (-0.3, 0.1) 0.32	
**Adjusted R**^**2**^	0.104		0.219	

β = Unstandardised regression coefficients

*statistically significant *P* < 0.05

CI: confidence interval

^***†***^*P* significance ≤ 0.15

PRTEE: Patient-Rated Tennis Elbow Evaluation, PFG: pain-free grip strength, HPT: heat pain threshold, PPT: pressure pain threshold, CPT: cold pain threshold, VDT: vibration detection threshold, LTT: longitudinal tendon thickness, TTT: transverse tendon thickness, TUSS: total ultrasound scale score

## Discussion

Structural changes in the load bearing tendon was previously conceptualised as the mechanism for pain in LE [16, 55]. However, contemporary literature suggests that structural changes in the affected tendon, such as hypoechoic and heterogeneous regions, bony abnormalities at the insertion, and neovascularity, do not correlate with the severity of clinical presentation [16, 17, 19]. While the non-association between tendon structure and clinical presentation is well documented, the relationship between tendon structure and sensory characteristics, and clinical presentation is less clear. The present cross-sectional study on 66 participants with a clinical diagnosis of LE provides unique insight into the interrelationship between US-observed structural changes, QST-measured sensory system changes, and clinical measures of pain and function. The results demonstrate that few tendon structural characteristics are related to sensory system changes, and in many cases the relationship is conflicting. For example, in the final model for VDT on the affected side, 22.5% of the variance could be explained by a combination of transverse tendon thickness and gender, with greater TTT and female gender associated with poorer VDT. In contrast, there was a significant inverse association between the absence of neovessels and higher (i.e. worse) CPT. Of the few structural or sensory characteristics that predicted clinical severity, greater HPT was the only significant variable associated with higher pain and disability (PRTEE) on the affected side, explaining 10.4% of the variance. For the unaffected side, only female gender predicted lower PFG, but greater TUSS predicted more impaired VDT.

The inverse relationships identified in this study, as reflected by more severe clinical symptoms associated with less severe structural and sensory characteristics, may be explained by Cook’s continuum model of pathology [13]. Pain in tendinopathy may be derived from the increased expression of nociceptive substances, stimulating the nociceptors near, or in, the peritendon. This nociceptive stimulation from the peritendon may occur due to increased tendon thickness, or via a reactive process in the healthy load-bearing portion of the tendon in response to the inability of the degenerative tendon portion to transmit tensile load [13]. This reactive-on-degenerative model may explain the lack of association between structural tendon changes and sensory and clinical characteristics in LE, as the size and severity of the degenerative tendon portion are not responsible for the magnitude of nociception in this model. This hypothesis requires further validation and may continue to evolve over time with the development of more sensitive imaging modalities.

Of interest is the significant side-to-side differences in sensory measures (PPT, CPT and HPT) in our LE population. Without a control group for comparison, we are unable to confirm the presence or absence of bilateral hyperalgesia. However, data extracted from Coombes et al. [27] suggests that CPT and HPT on the unaffected side in our LE cohort are not different to previously published control data (7.1 ±4.6°C and 44.3 ±2.5°C, respectively). As such, the unilateral mechanical and thermal (HPT and CPT) hyperalgesia in our population suggests a local peripheral pain state rather than a centrally-driven pain state. This is consistent with a recent study that found patellar and Achilles tendinopathies exhibited only local, rather than widespread, sensory changes [15]. This lack of bilateral hyperalgesia contrasts the findings reported by Coombes et al. [27] who found widespread cold hyperalgesia only in a severe subgroup of people with LE. A less severe clinical presentation in our cohort (PRTEE 30.8 compared to 40.1 in Coombes et al. [27]) may explain the lack of widespread changes identified in our study. We found that HPT was the only factor significantly associated with PRTEE, with higher HPT (i.e., less hyperalgesic) associated with higher pain and disability. This suggests that patients presenting to the clinic with high levels of pain and disability are likely to exhibit normal HPT, suggesting that the relevance of HPT in directing treatment is limited. Further, we observed that female gender was the only factor significantly associated with lower heat pain threshold, which is consistent with Rolke et al. [43].

Due to the high variability in QST measures *between* individuals, evidence suggests that side-to-side comparisons are more sensitive to change than between-group comparisons (e.g., against a control group) in detecting the gain (i.e., reduced threshold) or loss (i.e., increased threshold) of sensory function using QST [43]. As such, although the current study lacked a control group, the affected-to-unaffected side differences (i.e., relative reference data) in sensory measures from our study might be useful for clinicians to gain information about changes in sensory function in individuals with LE [43].

Participants showing increased transverse tendon thickness of the common extensor tendon in the affected elbows were more likely to have poorer vibration detection threshold (higher values indicates less sensitive VDT). The mean VDT (1.8 μm/sec) for affected elbows in our study was higher (i.e., poor detection of vibration) than age-matched normative values (ranging from 1.0 to 1.7 μm/s) previously reported [43, 56]. It may be that poorer VDT in the affected limb may occur through compression of the radial nerve via the larger cross-sectional area of the affected common extensor tendon. Recently, Gurcay et al. [57] reported the presence of increased thickness of the common extensor tendon with an increased cross-sectional area of the radial nerve on the affected side compared to the unaffected side in 44 participants with LE. Importantly, there was no loss of electrophysiological function on the affected side in this cohort, suggesting a subclinical picture of radial nerve entrapment may exist in some people with chronic LE. While our participants were screened for any neurological impairment, the elevated VDT may reflect a feature of subclinical impairment in sensory function that is not perceived by the individual. Elevated VDT (1.8 μm/sec) on the affected side in LE has previously been reported [24]. However, the difference in VDT in this previous study was not statistically significant when compared to a healthy control group, possibly due to the small sample size (LE group N = 11, Control N = 16) [24].

Neovascularity on the affected side was identified in only a small subgroup within our LE cohort (12/66). We found a negative association between the presence of neovessels and CPT, with the presence of neovascularity in the affected tendon associated with lower (i.e., less sensitive) CPT. That is, the absence of neovessels was associated with increased cold hyperalgesia on the affected side, which is consistent with previous work, which found the absence of neovessels was associated with a facilitated temporal summation of pain, lower pressure pain tolerance and higher habitual pain intensity [58]. Jespersen et al. propose that the presence of neovessels may reflect inflammatory activity associated with an early reactive overloading stage of LE, such as that described by Cook et al. [13]. This hypothesis assumes that neovessels disappear or reduce with chronicity of tendinopathy, a concept which has not yet been confirmed.

Although this cross-sectional study of 66 participants with LE provides insightful understanding of the interrelationship between tendon structural, sensory and clinical characteristics, there are some caveats and limitations to this study. Firstly, the cross-sectional study design and the absence of a control group limit our ability to assess the natural history of tendon structural and sensory changes in people with LE. Secondly, the presence of ultrasound detected tendon structural abnormalities and sensory changes in both affected and unaffected sides might be attributed to the normal ageing process [59], as the age range of participants in this study were 18 to 68 years. While we acknowledge that tendinopathic changes may be present in greater proportions in older age groups, the age of our study sample did not significantly influence the associations between tendon structural, sensory and clinical characteristics. Similarly, the US-observed tendon structural changes and the presence of sensory changes may have been compounded by the effects of previous corticosteroid injections. However, we found that only nine (13.6%) participants had a prior history of corticosteroid injection, and that there was no significant association between this history of corticosteroid injection, and tendon structural, sensory, or clinical measures. Finally, observer bias may have been present as a single assessor graded tendon abnormalities on static US images [60]. However, excellent inter-and intra-observer reliability in grading tendon abnormalities including using the US image rating scale was established prior to the current study and the assessor was blind to the clinical data during the US grading. In addition, the presence of intratendinous calcification was not included in the evaluation as the findings from a preliminary validation study revealed poor inter-rater reliability for the assessor in detecting the calcification.

## Conclusion

The results of this observational cross-sectional study indicate that structure and sensory measures were weakly associated with clinical characteristics in participants with chronic LE, reflecting a disconnect between structure, sensory and clinical presentation in LE. Notwithstanding this, increased transverse tendon thickness and neovascularity may be related to sensory system changes in LE.

## Supporting information

S1 Dataset (XLSX)
